# Serological evidence of chronic pulmonary *Aspergillosis * in tuberculosis patients in Kenya

**DOI:** 10.1186/s12879-022-07782-9

**Published:** 2022-10-25

**Authors:** Abdi Mohamed, Benear A. Obanda, Hannah K. Njeri, Sally N. Loroyokie, Olga M. Mashedi, Tom T. Ouko, Evangeline M. Gatumwa, Richard K. Korir, Takashi Yaguchi, Christine C. Bii

**Affiliations:** 1 Kenya Medical Research Institute, Centre for Microbiology Research, Mycology and Opportunistic Infections Laboratory, P.O.Box 54840-00200, Nairobi, Kenya; 2 Kenya Medical Research Institute, Centre for Respiratory Diseases Research, Tuberculosis Laboratory, P.O.Box 54840-00200, Nairobi, Kenya; 3 Medical Mycology Research Centre (MMRC), Division of BioResources, Chiba University, 1-8- 1 Inohana, Chuo-ku, 260-8673 Chiba, Japan

**Keywords:** Chronic pulmonary aspergillosis, Pulmonary tuberculosis, Resource limited settings, *Aspergillus* serology, Seroprevalence, Kenya

## Abstract

**Background:**

Pulmonary tuberculosis (PTB) is a significant risk factor for fungal infection. The cavitary lesions post PTB serves as a good reservoir for fungal colonization and subsequent infection. Furthermore, the severe immunosuppression associated with HIV and TB co-infection is another predisposition. The inadequate capacity to investigate and manage fungal infection in PTB patients increases their morbidity and mortality. The study aimed to provide serological evidence of chronic pulmonary aspergillosis (CPA) among PTB patients in Kenya. Towards this, we analysed 234 serum samples from patients presenting with persistent clinical features of PTB infections despite TB treatment in four referral hospitals.

**Methods:**

This was a cross sectional laboratory based study and patients were recruited following an informed consent. Serological detection of *Aspergillus fumigatus* IgG was done using enzyme-linked immunosorbent assay (Bordier Affinity Products SA). Sputum samples were subjected to microscopy and standard fungal culture. The isolated fungi were subjected to macro and micro morphological identifications and confirmed by sequence analysis of calmadulin, betatubilin and ITS genes.

**Results:**

Serological evidence of CPA or fungal sensitization was 46(19.7%) and equivocal or borderline was 14(6.0%). Mycological investigations of sputum resulted in 88(38%) positive for fungal culture. *Aspergillus* spp. accounted for 25(28%) of which *A. fumigatus* was 13(14.8%), *A. niger* 8(9.1%), *A. terreus*, *A. flav*us, *A. candidus* and *A. clavatus* 1 (1.1%) each. This was followed by *Penicillium* spp. 10 (11.4%), *Scedosporium* spp. 5 (5.7%) and *Rhizopus* spp. 3 (3.4%). Among the yeasts; *Candida albicans* accounted for 18(20.5%) followed by *C. glabrata* 5(5.7%). *Cryptococcus* spp. was isolated from 3(3.4%) of the samples while 13(14.8%) were other yeasts.

**Conclusion:**

Chronic pulmonary aspergillosis is a significant co-morbidity in PTB patients in Kenya that could be misdiagnosed as relapse or treatment failures in the absence of reliable diagnostic and clinical management algorithm. It could be the cause of persistent clinical symptoms despite TB treatment often misdiagnosed as TB smear/GeneXpert MTB/RIF® negative or relapse. We recommend that all patients with persistent clinical symptoms despite TB treatment should be subjected to fungal investigations before retreatment.

## Introduction

Chronic pulmonary aspergillosis is a progressively destructive lung disease caused by *Aspergillus* species, mainly *A.fumigatus.* The fungi can infect both immunocompetent and immunocompromised population [1]. The disease is common among individuals with previous or underlying pulmonary disorders such as tuberculosis, chronic obstructive pulmonary disease, sarcoidosis, atypical mycobacterial infection, emphysema and pneumothorax [2-4] and infrequently, among HIV, lung cancer and diabetes mellitus patients [2, 3].

Worldwide, CPA is estimated to affect about 3 million people, with associated high morbidity and mortality. Left untreated CPA mortality can be up to 80% within 5 years of infection [4, 5]. Pulmonary tuberculosis infection is recognised as the most important risk factor for the global burden of CPA. A recent global report estimates about 1.2 million patients with CPA as a sequel to PTB annually [6]. In developing countries with high TB burden, more than half of the cases of CPA are reported as a complication of post TB treatment. Pulmonary tuberculosis causes structural lung damages with residual cavitary lesions often present in 20–40% of patients. The cavitary lesions post TB infections serves as a good medium for infective fungal spore colonization preceding CPA progression [7, 8]. Unfortunately, most clinical and radiological features of pulmonary fungal infections mimic those of PTB or other pulmonary disorders. This results in misdiagnosis of PTB with patients being enrolled for retreatment without microbiological evidence [9].

Chronic pulmonary aspergillosis and PTB can co –exist, complicating diagnosis. In Africa and Asia, a co-infection rate of 15.4% among patients with PTB is reported. The two diseases are difficult to distinguish by clinical and radiological features alone[10, 11].

Sputum cultures are of low sensitivity with culture positivity of less than 80% of samples [4, 9, 12]. However, cultures of lung fluids of patients with CPA are usually positive for the infective *Aspergillus* species and other co-infecting pathogens [4, 8, 13]. Positive culture results are important for identifying the *Aspergillus* species causing infection and for performing drug susceptibility testing. However, fungal diagnostics are often not available in this settings a field that is somewhat ignored. Previous studies indicate misdiagnosis of CPA as PTB smear negative in 35% of patients due to fungal diagnostic challenges in resource limited settings [9, 12, 14].

Clinically, the Global Action Fund for Fungal Infections (GAFFI) defines CPA as an illness lasting 3 months with all of the following conditions: (1) weight loss, persistent cough, and/or haemoptysis; (2) chest images showing progressive cavitary infiltrates and/or a fungal ball and/or pericavitary fibrosis or infiltrates or pleural thickening and (3) a positive *Aspergillus* IgG assay result or other evidence of *Aspergillus* infection. This definition has been recommended for use in resource limited settings [9, 15].

Kenya ranks among the 30 high burden TB/HIV countries. As at 2020, the estimated TB incidence was 259 cases per 100,000 population [11]. Pulmonary TB is the fifth leading cause of death and a common complication of HIV infections in the country [16, 17]. While, PTB diagnosis has evolved considerably, there is unlikelihood that a MTB infected patient is misdiagnosed based on clinical, radiological, microscopy and now molecular assays including GeneXpert MTB/RIF®. Unfortunately, patients with worsening clinical symptoms but negative on MTB diagnostic algorithm are considered MTB relapse and retreated [11, 14, 16]. Approximately, 10% of TB sputum cultures reports fungal contamination [8, 18]. Unfortunately, fungi growing on TB cultures are usually considered culture contaminants without any consideration as a possible pathogen.

Due to diagnostic challenges of advanced imaging requirement, a positive *Aspergillus* IgG assay with clinical symptoms that mimic PTB relapse is a CPA case definition, after exclusion of alternate diagnoses (e.g. pulmonary TB or NTM). Such case is considered an evidence of *Aspergillus* infection and warrant fungal investigations and antifungal management in resource limited settings [9, 14]. Recently, a validated point-of-care lateral flow device, *Aspergillu*s IgG/IgM LFD (LDBio Diagnostic, Lyon, France) with a sensitivity and specificity of 91.6% and 98.0% respectively, was introduced for the rapid detection of *Aspergillus*-specific immunoglobulins [19]. However, the use of commercial enzyme-linked immunosorbent assay (ELISA) for detection of *Aspergillus* IgG serology has been reported to be superior in diagnosis of CPA and is considered the test of choice in clinical practice [20, 21].

Although the exact prevalence of CPA remains unclear in Kenya, a recent population based estimate put CPA burden following PTB infections at 10,848 cases with an incidence rate of 32 per 100,000 persons. However, this estimate is based on an actuarial approach and need to be treated with caution [22].

The lack of technical and infrastructural capabilities to diagnose and treat fungal infections and low index for clinical suspicion of CPA among clinicians, remains a key challenge in the diagnosis and management of respiratory fungal diseases. This is likely to result in significant morbidity and mortality among high risk patients in resource constrained settings. The study was aimed at providing serological evidence of CPA among PTB patients as a cause of smear/GeneXpert negative TB and /or TB treatment failures using A. fumigatus specific immunoglobulin G serology in four TB referral clinics in Kenya.

## Materials and methods

### Study design

A cross-sectional study was conducted between Jan 2019 to Dec 2019. The study population were adult patients with persistent clinical symptoms of pulmonary TB infections despite TB treatment in four TB treatment facilities in Kenya namely; Coast General Hospital (Mombasa county); Mbagathi Hospital (Nairobi county); Moi Teaching and Referral Hospital, (Uasin Gishu county) and Jaramogi Oginga Odinga Teaching and Referral Hospital (Kisumu county).

All patients were recruited following a written informed consent. Patients clinical/medical and sociodemographic data was obtained by resident chest physician through a structured questionnaire. Blood and fresh expectorated morning sputum samples were collected from all the study participants. The samples were transferred from the site hospitals in a cool box to the Mycology Reference Laboratory, KEMRI.

## Laboratory procedures

***Aspergillus*serology**.

Detection of *A. fumigatus* IgG antibodies was done using ELISA kit (Bordier Affinity Products SA). The procedures and results were done and interpreted according to the manufacturer’s protocol. The OD index of the sample was divided by the OD of the cut off serum. An OD index values of 1.0 and above were scored positive, values of 0.8 to 1.0 were considered equivocal or borderline, and values of 0.8 and lower were considered negative.

### Fungal culture

Sputum samples qualified as per Murray-Washington’s score (> 25 polynuclear leukocytes and < 25 squamous epithelial cells per low-power field) were used [23]. Samples were subjected to direct microscopy and standard fungal culture [24]. Briefly, sputum samples were first mucolysed in dithiothreitol (Sputasol® Oxoid, UK) as previously described [25] and then divided into two parts, one for fungal culture and other for microscopic examination. Samples for fungal culture were inoculated on sabouraud dextrose agar plates (Oxoid, UK) supplemented with chloramphenicol at concentration of 0.5 mg/mL to inhibit bacterial contamination. The fungal cultures were incubated at 35^o^C for 4 weeks and evaluated daily for fungal growth. Samples for microscopic examination were mounted with 20% potassium hydroxide. All cultures were performed in duplicates.

### Fungal identification

Yeasts isolates were differentiated on CHROMagar® Candida and corn meal agar. Casein agar was used for differentiation of *Candida albicans* and *Candida dubliensis.* Preliminary identification of *Cryptococcus* spp. was done using urea hydrolysis and corn meal agar. Confirmations of yeasts were done using the Analytical Profile Index (API) 20 C aux (BioMerieux® SA).

Moulds identification was done using colony macroscopic and microscopic morphological features as described by Larone et al. 2018 [24]. Macroscopic features such as colony growth rate (colony diameter) colour of surface and reverse, texture, diffusing pigment and temperature tolerance were used. Microscopic feature on Lactophenol cotton blue stain at x40 magnification such as; conidia, shapes and arrangement of phialides were used. Confirmations of the isolates were done by sequence analysis of β-tubulin, Calmodulin, and ITS genes courtesy of Prof. Yaguchi of the Medical Mycology Research Institute-Chiba University-Japan.

### Data analysis

Data was collected using a structured questionnaire and results of serological tests were entered into MS-Excel (2010). Statistical analysis was done using SPSS software version 18.0. Descriptive statistics were presented as percentages. Pearson chi-square (*x*^2^) test was used for test of association between categorical variables. Significant deference was determined using a Z test and a P-value of < 0.05 considered significant.

## Results

### Enzyme immunoassay for diagnosis of *Aspergillosis*

The serological prevalence of *Aspergillosis* from a total of 234 serum samples from the study sites was 19.7% with an overall mean titres of 1.07. The Nairobi study site had the lowest seroprevalence of A.fumigatus IgG antibody 6 (13.3%) compared to Mombasa 15 (20%), Kisumu 6 (21.4%) and UasinGishu 19 (22.1%). However, A. fumigatus IgG seroprevalence was not significantly different with region, x^2^ = 1.524;p = 0.68.

Mombasa had the highest equivocal scored titres of 10.7% compared to < 5% of all the other sites. In western Kenya, Uasin Gishu had the lowest mean tires of 0.83 and Nairobi with the highest mean titres of 1.35. The regional distribution of *A. fumigatus* IgG seroprevalence is shown in (Table 1).

**Table 1:** Serological *A. fumigatus* IgG levels in different regions of Kenya

County(N)	Study sites					Mean titre index	*P* value
			**Positive**	**Equivocal**	**Negative**		
**Nairobi (45)**		MBH	6/45(13.3)	2/45(4.5)	37/45(82.2)	1.35	**0.68**
**Mombasa (75)**		CGH	15/75(20.0)	8/75(10.7)	52/75(69.3)	1.11	
**Kisumu (28)**		JOOTRH	6/28(21.4)	1/28(3.6)	21/28(75.0)	0.97	
**UasinGishu (86)**		MTRH	19/86(22.1)	3/86(3.5)	64/86(72.5)	0.83	
		**Total**	**46/234(19.7)**	**14/234(6.0)**	**174/234(74.4)**	**1.07**	

Key: MBH-Mbagathi Hospital, CGH- Coast General Hospital, JOOTRH- Jaramogi Oginga Odinga Teaching and Referral Hospital and MTRH- Moi Teaching and Referral Hospital. Data are represented as number (%)

61% of the seropositive cases were female and only 39% were of male gender while 18–40 years’ age bracket constituted the majority 54.3%. The ages of the participants ranged from 5 to 92 years, with a mean age ± SD of 41.55 ± 16.07 years, a median and mode of 41 and 27 years respectively.

Self-employment was 52.2% among the seropositive individual with only 19.6% in formal employment. The unemployed were 21.7% among the population studied. Evidence of fungal spores exposure through leaking or mouldy houses was reported in 47.8% of the population while only 23.9% ever smoked or were passive smokers (Table 2). .

**Table 2:** Social demographics features of *A.fumigatus* IgG positive and negative individuals

Demographics		IgG positive N = 46	IgG negative N = 188	*P* value
**Gender**	F	28(60.9)	127(67.6)	0.00
	M	18(39.1)	61(32.4)	
**Age**	18–40	25(54.3)	90(47.9)	0.68
	41–60	15(32.6)	74(39.4)	
	> 61	6(13.1)	24(12.8)	
	Employed	9(19.6)	44(23.4)	0.90
**Occupation**	Self	24(52.2)	94(50)	
	Not employed	10(21.7)	35(18.6)	
	Student	3(6.5)	15(8)	
**Habitation**	Moldy or leaking house Non	22(47.8) 24(52.2)	30(16) 158(84)	0.00
**Smoking**	Y/Ps	11(23.9)	15(8)	0.00
	No	35(76.1)	173(92)	

Key: Y- ever smoked, Ps- passively smoked, data are represented as number (%)

Productive cough 87%, night sweat 71.7%, fever and chest pains 69.6% were most common in *A. fumigatus* IgG seropositive individuals. Bloody cough was present in only 19.6% of the individuals while shortness of breath and fatigue were present in 54.3% and 43.5% respectively. Majority, 87% of the cases were GeneXpert negative while only 4.3% were positive and only 8.7% were not determined. Majority, 47.8% of the patients were cases of worsening symptoms despite TB treatment while 28.3% were new cases and 23.9% were not on any treatment despite meeting PTB clinical criteria (Table 3).

**Table 3:** Diagnosis and clinical features of A. *fumigatus* IgG positive individuals

		Present	Absent	*P* value
**Clinical features**	Productive cough	40(87)	6 (13)	0.89
	Bloody	9(19.6)	37(80.4)	0.46
	Fever	32(69.6)	14(30.4)	0.99
	Night sweat	33(71.7)	13(28.3)	0.78
	Chest pain	32(69.6)	14(30.4)	0.99
	Shortness of breath	25(54.3)	21(45.7)	0.04
	Tired	20(43.5)	26(56.5)	0.42
	** N = 46**			
**GeneXpert status**	Positive	2(4.3)		-
	Negative	40(87.0)		
	ND	4(8.7)		
	**N = 46**			
**TB treatment**	New TB	13(28.3)		-
	Symptoms despites treatment	22(47.8)		
	None	11(23.9)		
	**N = 46**			

ND-not done; data are represented as number (%)

### Mycological investigations

Out of 234 sputum samples cultured, 88 (38%) were positive for fungi. Filamentous fungi and yeasts are the most common isolates 49 (55.7%) and 39 (44.3%) respectively. Among the filamentous fungi, *Aspergillus* spp. formed the majority 25 (28%). Of the 25 *Aspergillus* spp. isolates, *A. fumigatus* was the most isolated 13 (14.8%), followed by *A. niger* 8 (9.1%), *A. terreus*, *A. flavus, A. candidus*, and *A. clavatus* with 1 (1.1%) each. *Aspergillus* spp. was followed by *Penicillium* spp 10 (11.4%), *Scedosporium* spp. 5 (5.7%) and *Rhizopus* spp. 3 (3.4%). Among the yeasts isolated were *Candida albicans* 18 (20.5%) while non albicans *Candida* (NAC) was 17 (19.3%). Among the NAC isolated were, *C.glabrata 5* (5.7%), *C. Krusei* 4 (4.6%), C. *tropicalis* 3 (3.4%), while *C. parapsilosis* and *C. zeylanoich* was isolated at 2 (2.3%) each. *Cryptococcus* species and *Geotrichum candidum* was isolated in 3 (3.4%) and 1(1.1%) of the samples respectively. There were mixed fungal elements detected in some cultures (Table 4).

**Table 4:** Mycological findings of sputum culture from 88 positive cases of patients attending the pulmonary tuberculosis clinics

Fungal isolate	Frequency	Percent
*C.albicans*	18	20.5
* A.fumigatus*	13	14.8
*Penicillium spp*	10	11.4
* A. niger*	8	9.1
*Scedosporium spp.*	5	5.7
* C. glabrata + C.albicans*	5	5.7
* C. Krusei + C.glabrata*	4	4.6
*Rhizopus spp.*	3	3.4
* C. tropicalis*	3	3.4
*Cryptococcus spp.*	3	3.4
*Phiallophora spp.*	2	2.3
*Cladosporium spp.*	2	2.3
* C. parapsilosis*	2	2.3
* C. zeylanoich*	2	2.3
* A.terreus*	1	1.1
* A.flavus*	1	1.1
* A.candidus*	1	1.1
* A.clavatus*	1	1.1
*Ochroconis spp.*	1	1.1
*Scapulariopsis brevicaulis*	1	1.1
* C. guilliamondii*	1	1.1
*Geotrichum candidum*	1	1.1
**Total**	**88**	**100**

## Discussion

Tuberculosis remains a significant cause of death and a major public health problem worldwide. In 2021, a WHO report indicates that 10 million people were infected with TB with 1.5 million deaths. In Africa, TB accounts for about 80% mortality among people living with HIV/AIDS [11, 26]. Chronic Pulmonary Aspergillosis is a well-established complication of PTB [5, 8, 9, 26, 27].

The current study evaluated current and post pulmonary tuberculosis patients for CPA as a cause of smear/GeneXpert negative TB and /or TB treatment failure using sputum fungal culture and A. fumigatus specific immunoglobulin G serology in four TB referral clinics in Kenya. The overall seroprevalence of A. fumigatus IgG antibody among patients in our setting was 19.7%. This finding is comparable with findings from similar studies in Nigeria 14.5% [20], Iran 13.7% [28] and Japan 16.7% [29]. In the Iran and Nigeria studies, HIV infected PTB patients were excluded in the analysis due to a markedly lower CPA seroprevalence rates compared to HIV negative patients. The low CPA seroprevalence among HIV positive patients is thought to possibly be due to poor immune response during *Aspergillus* infections in this population, particularly in patients with low CD4 counts [20].

In 2020, Kenya reported overall tuberculosis infections of 140,000 patients, of which 120,400 (86%) were pulmonary tuberculosis cases with 6.3% mortality among PTB [17]. If we apply the CPA seroprevalence rate obtained in this study, we could anticipate an annual incidence of approximately 22,225 CPA cases among pulmonary tuberculosis patients. This is substationally greater than the population estimate of 10,848 CPA cases [22]. This finding indicates that CPA is a significant co-morbidity in PTB patients in our setting and warrant fungal investigations.

We analysed specific antibodies against *A. fumigatus*, because, it is the most common fungi implicated in chronic pulmonary infections worldwide. In addition, A*spergillus* IgG is particularly sensitive and positive in over 90% of patients with CPA [1, 19, 21, 26]. However, other *Aspergillus* species including *A. niger*, *A. flavus* have been detected in CPA patients. Antibody assays for *A.fumigatus* may have low sensitivity for detection of infection with non *fumigatus Aspergillus*. According to Oladele and co-workers, ImmunoCAP® automated system (ThermoFisher Scientific, USA) was unable to detect *A*.niger and *A.flavus* species in CPA confirmed positive sputum cultures [20]. In our study, a significant proportion, 74.4% of the population were *A.fumigatus* IgG negative. In this population, twelve sputum samples obtained were positive for non *fumigatus* pathogens, with predominantly *A.niger* species, 9.09%. Our findings is comparable to findings from other studies among CPA patients in which *A.niger*, was second most isolated fungi from sputum cultures [18, 20, 28, 30].

The ELISA antibody assay (Bordier Affinity Products SA) used in this study has sensitivity and specificity of 97% and 90.3% respectively. It has superior performance compared to other commercial ELISA *A.fumigatus* IgG tests and only comparable to automated fluorescent ELISA assays [21, 31].

Due to low sensitivity of sputum culture and serology [9, 21] it is likely that the actual burden of pulmonary aspergillosis among pulmonary tuberculosis patients in our settings is under represented, considering that over 40% of the clinical Aspergillus species are non fumigatus Aspergillus. Aspergillus niger, A. terreus and A. flavus are common human pathogens while A.clavatus and A. candidus and other species are known potent allergens [32].

Geographical and climatic variation in *Aspergillus* IgG seroprevalence and fungal recovery has been reported elsewhere [28, 33]. In the present study, Nairobi county, a warmer region with annual temperature average of 18.8 °C reported low seroprevalence compared to the other regions in the study with higher average annual temperatures [34].

In the present study, more female than male patients were seropositive for *A.fumigatus* IgG antibody

and reported worsening clinical symptoms. There was a significant difference between the two groups, Z = 3.457; *p* = 0.00, with females 3 times likely to test positive for *A.fumigatus* IgG antibodies than males (OR 3.24; 95% CI 1.664–6.305) (Table 2). In a similar study in Taiwan, being female gender and previous pulmonary TB were found to be independent predictors of *A.fumigatus* IgG positivity [33]. In addition to gender, mouldy habitation and smoking were important demographic risk factors for *A.fumigatus* IgG seropositivity, *p* = 0.00 and *p* = 0.00 respectively. However, age and occupation did not show significant association with *A.fumigatus* IgG seroprevalence (Table 2).

The most common clinical features of the patients positive for *Aspergillus* infections were productive cough, fever, chest pain and night sweat and less frequently haemoptysis. These symptoms are consistent with previous studies and suggest an acute clinical presentation due to chronic pulmonary aspergillosis and/or coinfection with bacteria or other pathogens [10, 14, 35, 36]. There was a significant difference in clinical presentations between the gender, Z = 2.239; *p* = 0.01, with *A.fumigatus* IgG seropositive females 4 times likely to experience difficulty in breathing than males (OR 4.22; 95% CI 1.197–14.896) (Table 3).

About 20% of patients positive for *A.fumigatus* specific IgG antibodies presented with haemoptysis (bloody sputum). Haemoptysis is a well know presentation of pulmonary aspergilloma and is associated with high morbidity and mortality [20, 37, 38]. In a study in Nigeria, aspergilloma associated haemoptysis accounted for over 30% mortality, despite surgical resection and treatment [39]. Implying that, early diagnosis and treatment is crucial to improve patient outcomes, prevent disease progression and lower morbidity and mortality.

Kenya introduced GeneXpert MTB/RIF® in 2011, as a presumptive diagnostic test for suspected TB infections. However, smear microscopy is still the gold standard used in many peripheral health facilities where GeneXpert® is not available. In both cases, conventional CXR is often the initial imaging method for evaluation of pulmonary infections [9, 14, 40]. However, conventional CXR is of low sensitivity and specificity [10]. In the absence of advanced imaging techniques in resource limited settings, a positive *Aspergillus* IgG assay with CPA case definition after exclusion of alternative diagnoses (e.g., pulmonary TB or NTM) is an important evidence of *Aspergillus* spp. infections in patients misdiagnosed with PTB in Uganda [9, 14].

Nearly half, 47.8% of patients were being managed for PTB disease despite GeneXpert negative results. This finding is comparable with findings from similar studies in which 35% of CPA patients were misdiagnosed and managed with anti-TB medication based on clinical suspicion without evidence of mycobacterium infection [12]. In this settings, patients negative on TB diagnostic algorithms and presenting with PTB like symptoms are often considered TB relapse and retreated [14, 17, 20].

In our study sites, no laboratory was performing fungal culture or *Aspergillus* serology for patients with clinical symptoms that mimic PTB relapse following GeneXpert negative results, neither were patients on antifungal treatment. Lack of diagnostic capacity for fungal infections and low index of suspicion for fungal respiratory infections among clinicians is thought to be a challenge to CPA diagnosis and management in resource limited settings [7, 41].

Mycological investigations of sputum samples confirmed fungi with potential to cause respiratory infections and invasive disease particularly among individuals with immunosuppression and those with respiratory disorders [7, 26]. Previous study in Kenya by Tonui and co-workers [30] isolated pathogenic and opportunistic fungi from sputum samples of smear negative PTB retreatment patients attending TB clinics. These studies highlight the significance of pulmonary fungal colonization and infection among bacteriolocally negative patients presenting with clinical symptoms suggestive of pulmonary TB. Microbial colonization of respiratory tract has been associated with poor treatment outcomes among PTB patients [18, 42].

In the present study, nearly half 47.8% of patients positive for *A.fumigatus* IgG, reported exposure to fungal spores through mouldy habitation. These contaminated indoor and outdoor environments [22, 43] serve as a source of inhalation of infective fungal spores and yeasts and may explain the diversity of fungi recovered from the patient’s sputum.

Management of CPA requires long term antifungal therapy of over 6 months, essentially to prevent haemoptysis and relapse [7, 44, 45].Therefore, early diagnosis and treatment is important to lower morbidity and hence reduce the apparent high mortality for PTB patients in the country.

The national diagnostic and treatment algorithms for GeneXpert negative patients with PTB symptoms are not clear. To our knowledge, no study has been done to evaluated CPA incidences or seroprevalence among pulmonary TB patients in Kenya.

The study excluded patients with extra pulmonary TB and those on second line anti-TB treatment. The HIV status of the patients were also not done, which could introduce some bias and study limitation.

## Conclusion

Our findings suggest that CPA is common in GeneXpert MTB/RIF® negative patients and could be the cause of persistent clinical symptoms in PTB patients in our settings. In the absence of a reliable testing capacity, CPA may be misdiagnosed as PTB relapse or treatment failures with patients unnecessarily put on anti TB medication for extended periods with serious health consequences. We recommend that patients with persistent clinical symptoms despite TB treatment should be considered for specific *Aspergillus* antibody testing for CPA and for microbiological investigations for a definitive diagnosis before retreatment.

## Data Availability

The datasets generated and/or analyzed during the current study are not publicly available due to patients confidentiality but are available from the corresponding author on reasonable request.

## Abbreviations

AIDS: Acquired Immunodeficiency Syndrome
CPA: Chronic Pulmonary Aspergillosis
HIV: Human Immunodeficiency Virus
IgG/IgM: Immunoglobulin G/M
IRG: Internal Research Grant
ITS: Internal Transcribed Spacer
KEMRI: Kenya Medical Research Institute
LFD: Lateral Flow Device
MMRC: Medical Mycology Research Centre, Japan
MTB: Mycobacterium Tuberculosis
NTM: Non Tuberculous Mycobacterium
OD: Optical Density
PTB: Pulmonary Tuberculosis
WHO: World Health Organization
